# Galectin-1: A Traditionally Immunosuppressive Protein Displays Context-Dependent Capacities

**DOI:** 10.3390/ijms24076501

**Published:** 2023-03-30

**Authors:** Xizhi Yu, Junjie Qian, Limin Ding, Shengyong Yin, Lin Zhou, Shusen Zheng

**Affiliations:** 1 Division of Hepatobiliary and Pancreatic Surgery, Department of Surgery, The First Affiliated Hospital, School of Medicine, Zhejiang University, Hangzhou 310003, China; 2 NHFPC Key Laboratory of Combined Multi-Organ Transplantation, Hangzhou 310003, China; 3 Key Laboratory of the Diagnosis and Treatment of Organ Transplantation, CAMS, Hangzhou 310003, China; 4 Key Laboratory of Organ Transplantation, Hangzhou 310003, China; 5 Collaborative Innovation Center for Diagnosis Treatment of Infectious Diseases, Hangzhou 310003, China

**Keywords:** galectin-1, inflammation, tumor microenvironment, immune regulation, treatment

## Abstract

Galectin–Carbohydrate interactions are indispensable to pathogen recognition and immune response. Galectin-1, a ubiquitously expressed 14-kDa protein with an evolutionarily conserved β-galactoside binding site, translates glycoconjugate recognition into function. That galectin-1 is demonstrated to induce T cell apoptosis has led to substantial attention to the immunosuppressive properties of this protein, such as inducing naive immune cells to suppressive phenotypes, promoting recruitment of immunosuppressing cells as well as impairing functions of cytotoxic leukocytes. However, only in recent years have studies shown that galectin-1 appears to perform a pro-inflammatory role in certain diseases. In this review, we describe the anti-inflammatory function of galectin-1 and its possible mechanisms and summarize the existing therapies and preclinical efficacy relating to these agents. In the meantime, we also discuss the potential causal factors by which galectin-1 promotes the progression of inflammation.

## 1. Introduction

There is a coat with abundant glycoconjugates on the cell and microbe surface [1]. The glycosylation of these compounds is a complex process with carbohydrates attached to the hydroxyl group of serine or threonine (O-glycan) and carbohydrates attached to the nitrogen of asparagine (N-glycan). Indeed, diverse signals can be transmitted with different glycosylation states [2]. Thus, deciphering the biological information encoded by glycan has become a promising field for revealing novel mechanisms of physiological and pathological processes.

The proteins characterized by their high affinity for β-galactosides and evolutionarily conserved carbohydrate recognition domain (CRD) were first named “galectins” in 1994 [3]. In contrast to other chemokines, cytokines, or transcription factors, galectins bind to the outermost grouping of carbohydrates on a glycoprotein or glycolipid oligosaccharide rather than specific receptors [1], thus mediating a range of vital activities. To this date, 15 members of galectins have been identified in mammals and are able to be classified into three categories according to their structure: prototype with one CRD, tandem repeat-type with two CRDs, and chimeric-type forming oligomers [4]. Among them, 11 galectins are found in humans, and some of them are widely expressed in a wide range of tissues (such as galectin-1 and -3), whereas other galectins display tissue specificity (galectin-4 and -10) [5]. The expression of these proteins can be regulated during organ and tissue development and the differentiation of specific cells [6]. After synthesis, galectins can be found both extracellularly and intracellularly. Even though there is no classical signal sequence at the N-terminus, it can be secreted via an unconventional protein secretion pathway, meaning that protein secretion does not occur via the ER-Golgi complex [7]. Extracellular galectins are capable of binding to suitable glycoconjugates on the cell surface, extracellular matrix, and even on the surface of pathogens [8,9]. By contrast, Intracellular galectins are involved in various cellular activities including pre-mRNA splicing, cell-cycle progression, cell growth, and apoptosis, through the protein-protein interaction rather than galectin-carbohydrate interaction [6,10,11].

Of note, high expression of galectins is typically present at sites of inflammation and in the microenvironment of cancer, suggesting an intimate relation of galectins to immunity. Indeed, galectins exert diverse roles in pathogen recognition, antigen processing, leukocyte trafficking, immune activation, and suppression [1]. However, only galectin-1 and galectin-3 have undergone a detailed study and demonstrated to be effective for further applications, whereas the biological functions and mechanisms of other galectins still expect deep investigations [12]. Here, we describe in detail the anti- as well as the pro-inflammatory function of galectin-1 in immune response and show that these seemingly paradoxical effects are dictated by a body of contributing factors.

## 2. Molecular Structural and Biological Function of Galectin-1

Galectin-1 is a 14-kDa protein that contains 135 amino acids and is encoded by the *LGALS1* gene [13]. Human galectin-1 is soluble and exists in a dimeric form maintained by non-covalent binding [14]. This dimeric protein is composed of the 22-strand anti-parallel β-sandwich, and each monomer contains a CRD [15]. In addition to being found in the cytoplasm, galectin-1 is also found to be present on the cell membrane and can be secreted into the extracellular matrix. Of note, each monomer of galectin-1 contains six cysteine residues (Cys2, Cys16, Cys42, Cys60, Cys88, and Cys130), and the reduced or oxidized states of them play a significant impact on the function of this protein [16]. The oxidation of galectin-1, existing as a monomer, reduces the T-cell apoptosis activity [16] but shows an ability to promote axonal regeneration [17].

Galectin-1 is broadly expressed in a wide range of tissues as well as cell types and can exert its effects both intracellularly and extracellularly [18]. It was reported that intracellular galectin-1 is not only a functionally redundant splicing factor, which can bind splicing partners through weak protein-protein interactions [19] but is also involved in intracellular signaling [20]. Extracellular galectin-1 typically exists in the reduced state and performs function through carbohydrate recognition domains. By forming cross-linking heterodimers, extracellular galectin-1 can facilitate interactions between cells and cells as well as cells and extracellular matrix [21,22]. Interestingly, secreted galectin-1 bound to the cell surface or extracellular matrix has a more substantial effect than soluble galectin-1, as Jiale He et al. found that galectin-1 on Matrigel could kill T cells at Ten-fold less concentration than soluble galectin-1 [23]. Since its discovery, galectin-1 has been demonstrated to mediate diverse physiological and pathological processes, such as being involved in cell growth and migration, inflammation, angiogenesis and promoting nervous system development, muscle differentiation, and tumor progression, mediating evasion of cancer immune surveillance, immune tolerance in the early pregnancy and cell adhesion [18,24,25,26]. In recent years, there has been growing awareness that galectin-1 plays a vital role in modulating immune response. In this case, we attempt to shed light on the effect of galectin-1 in infection, transplantation, tumor, and autoimmunity-related diseases.

## 3. Galectin-1 Functions as a Context-Dependent Regulator in Infection

It is often considered that galectin-1 performs an anti-inflammatory role in most cases, as Rabinovich et al. demonstrated that bee venom phospholipase A(2) induced acute inflammation was attenuated by galectin-1 in the rat hind paw edema test [27]. However, this may only sometimes be the case since the effect of galectin-1 may be changed by the stage of inflammation, the status of cell glycosylation, and many other factors [28,29]. The dual roles of galectin-1 in infection are discussed in the following three sections.

### 3.1. Bacterial Infection

In most instances, bacteria can take advantage of the anti-inflammatory effects of galectin-1 to circumvent protective host immunity. In the research by Davicino et al., endogenous galectin-1 regulate tolerogenic response by impairing the production of interferon-γ (IFN-γ) and interleukin (IL)-17, repressing synthesis of tumor necrosis factor (TNF) and nitric oxide (NO) as well as activation of nuclear factor kB (NF-kB), thereby promoting infection of *Yersinia enterocolitica* [30]. Interestingly, in the recent research of this same microorganism, Jofre et al. proposed a novel mechanism that galectin-1 could bind to virulence factors of *Yersinia enterocolitica* named Yops and protect them from trypsin digestion [31]. For intracellular bacteria such as *Tropheryma whipplei*, which can replicate in macrophages, crosslinking between bacterial glycans and cell surface glycans mediated by galectin-1 facilitate *T. whipplei* cell entry [32]. Of note, the anti-inflammatory effects of galectin-1 do not always play the role of “evildoer” in bacterial infection. It was reported that galectin-1 substantially attenuated CD4+ T cells, neutrophils, and CD45+ T infiltration as well as T helper (Th) 17 response, which diminished severe corneal immunoinflammatory impairment caused by infection of *Pseudomonas aeruginosa* [33].

However, galectin-1 also exerts pro-inflammatory effects in certain cases and promotes inflammatory lethality. Such dual effects appear to be most pronounced in neutrophils. It was shown that galectin-1 was capable of inducing phosphatidylserine (PS) exposure on the surface of human-activated, rather than resting, neutrophils, which promoted their phagocytosis by activated macrophages [34,35]. Nevertheless, Almkvist et al. demonstrated that galectin-1 contributed to the activation of the NADPH-oxidase in primed neutrophils [36]. These seemingly paradoxical roles of galectin-1 may be a protective mechanism that strengthens the bacterial killing capacity of neutrophils while protecting healthy tissues from inflammatory damage. However, the reactive oxygen species (ROS) production was not enhanced in naïve neutrophils following galectin-1 induction and pretreatment with galectin-1 attenuated the production of ROS upon stimulation of N-formyl-methionyl-leucyl-phenylalanine (fMLP) and phorbol myristate acetate (PMA), suggesting that the role of galectin-1 depended on the activation state of neutrophils as well as the stage of the inflammatory response [36,37]. Additionally, through binding to sialoglycoprotein CD43, galectin-1 was evidenced to induce the migration of human resting neutrophils under physiological conditions without additional inflammatory insults, whereas inhibition of polymorphonuclear leukocyte migration was observed following treatment with galectin-1 for 4 h in a murine acute inflammation model, accompanied by impaired expression of adhesion molecules [38,39]. Moreover, an updated study demonstrated that galectin-1 was an inflammatory damage-associated molecular pattern (DAMP) whose release was elicited by cytosolic lipopolysaccharide (LPS) sensing during Infections caused by Gram-negative bacteria. Additionally, it could accentuate lethal inflammation caused by sepsis through the inhibition of CD45 [28].

Overall, on the one hand, galectin-1 facilitates bacterial infection via diminishing the host immune response, protecting bacterial causative proteins as well as mediating the transport of bacteria. On the other hand, it is capable of promoting the development of inflammation in specific circumstances by enhancing the killing ability of neutrophils and augmenting inflammatory damage elicited by bacterial endotoxins.

### 3.2. Viral Infection

Mounting evidence indicates that galectin-1 performs a number of functions via multiple mechanisms during virus infection. Take HIV infection as an example, galectin-1 was proved to facilitate interactions between viral envelope gp120 and host CD4+ T lymphocytes, thereby promoting attachment of the virus to target cells [40]. In addition to CD4+ T lymphocytes, monocyte-derived macrophages, which are usually one of the first target cells encountered by the virus, also provide a cellular environment for viral replication [41]. Additionally, galectin-1 mediates virus adhesion to macrophages in a glycan-binding manner [41,42]. As for the Nipah virus (NiV), it was reported that galectin-1 promoted NiV attachment to human epithelium [43].

However, during infection of the Dengue virus, galectin-1 seems to perform an anti-infection role. Toledo et al. found that galectin-1, rather than galectin-3, directly binding to dengue virus type 1 (DENV-1) causes inhibition of its internalization and adsorption to host cells, instead of facilitating adhesion [44]. Similarly, the infectivity and hemagglutination activity of the influenza virus was also diminished following galectin-1 binding to the viral envelope. Additionally, galectin-1 was applied as an intranasal treatment to attenuate inflammation, viral load as well as cell apoptosis caused by influenza in the lung [45]. Moreover, galectin-1 can bind to NiV-F and NiV-G, specific envelope glycoproteins of the Nipah virus, thereby thwarting endothelial cell fusion and syncytia formation [46,47]. This implies that galectin-1 is a protective factor during NiV infection, which seems to contradict what was mentioned above. The study of Garner et al. may explain this contradiction [43]. They found that the timing of virus exposure to galectin-1 could alter the effects. That is, pre-infective galectin-1 promotes NiV infection; in contrast, post-infective galectin-1 impairs syncytium formation as well as virus production, and this inhibition is unique to the *Paramyxoviridae* family [43,47]. These results suggest that the dual effects of galectin-1 are context-dependent and determined by multiple factors including species of virus and timing of exposure.

### 3.3. Parasitic Infection

In parasitic infections, galectin-1 usually plays a pro-infection role. Endogenous galectin-1 was demonstrated to be a facilitator of parasitic infection by Poncini et al. in a *Trypanosoma cruzi* infection model. They found that a deficiency of galectin-1 thwarted the activation of dendritic cells (DCs) and regulatory T cells (Tregs), which meant galectin-1 fueled the immunotolerant circuits [48]. As for exogenous galectin-1, this glycan-binding protein secreted by *Angiostrongylus cantonensis* could bind to Annexin A2 and induce macrophage apoptosis [49]. In addition to exerting immunosuppressive properties, galectin-1 also mediates interactions between parasites and hosts. In the research of Okumura et al., they found that *Trichomonas vaginalis* were covered with lipophosphoglycan and contained a high abundance of galactose, which could serve as a binding site for galectin-1. Therefore, this parasite could attach to host cells through glycoconjugates [50]. Similarly, Petropolis demonstrated that human galectin-1 mediated the adhesion of *Entamoeba histolytica* to host endothelial cells in an in vitro human 3D-liver model [51]. Thus, the pro-infection abilities of galectin-1 are twofold. Firstly, both endo- and exogenous galectin-1 display immunosuppressive capability. Secondly, galectin-1 induces host-parasite interactions.

## 4. Galectin-1: A “Guardian” of Allogeneic Graft

The success of allogeneic transplantation is determined by numerous factors. Among them, a delicate immune balance between graft and host performs a significant role. Strong host immune defense causes graft necrosis, whereas excessively powerful anti-host reaction leads to graft versus host disease (GVHD). GVHD occurs when the host immune system is inhibited and donor T cells respond to host self-antigens, so it tends to develop after allogeneic hematopoietic stem cell transplantation [52]. Galectin-1, generally considered an immunosuppressive molecule, was demonstrated to improve survival after transplantation and attenuate graft versus host immunity by Baum et al. They found that the production of IL-2 and IFN-γ was diminished, and cellularity in the spleen and bone marrow was increased in galectin-1-treated transplanted mice [53]. Moreover, human mesenchymal stromal cells were demonstrated to secret galectin-1 to regulate the release of GVHD-related cytokines including IL-10, IL-2, TNF-α as well as IFN-γ [54].

In addition to inhibiting graft versus host immune response, galectin-1 can also attenuate graft rejection. As the primary effector cell in host-versus-graft immune defense, human T lymphocytes stimulated with allogeneic cells from donors have been approved to be inhibited by galectin-1 in vitro, which was accompanied by Bcl-2 downregulation and caspase activation [55]. For further verification of the protective effects of galectin-1, a rat allogeneic renal transplantation model was established by Xu et al. They found that galectin-1 injection could reduce serum concentrations of IFN-γ and CD30 and decrease the percentage of CD8+ T cell subset, thereby extending the survival of recipient animals [56]. Similarly, in the research of Moreau et al., endogenous galectin-1 reduced the IL-17 and IFN-γ secretion by CD8+ T lymphocytes and the percentage of this subset, resulting in a difference in survival of the mouse skin transplantation model [57]. As for liver transplantation, galectin-1 has also been proved as a protective factor, prolonging the survival of fms-like tyrosine kinase 3 ligand pretreated mouse liver allograft [58]. Moreover, recent studies found that galectin-1 produced by Tregs and hepatic stellate cells exerted a significant role in inducing immune tolerance following transplantation [59,60]. Interestingly, galectin-1-induced tolerogenic dendritic cells were shown to control graft rejection as well. Peng et al. found that apoptotic lymphocytes infusion combined with galectin-1-induced DCs significantly prolonged allograft survival [61]. Overall, galectin-1 performs a vital role in maintaining host as well as graft immune tolerance, providing a novel treatment for transplant rejection.

## 5. Cancer Cells Hijack Galectin-1 to Evade Immune Surveillance

**Figure 1:**
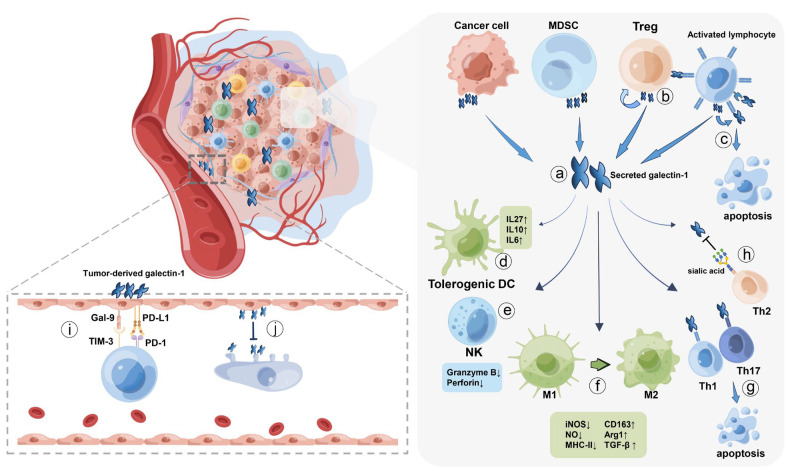
The roles of galectin-1 in the tumor microenvironment. (a) Galectin-1 was reported to be secreted by cancer cells, myeloid-derived suppressor cells (MDSC), regulatory T cells (Treg), and activated T lymphocytes in the tumor microenvironment. (b) Galectin-1 is able to promote the expansion of Treg cells and act as a mediator maintaining the immunosuppressive property of Treg cells. (c) In addition to inducing apoptosis of activated T lymphocytes, galectin-1 also acts as a negative autocrine regulator. (d) Galectin-1 in the tumor microenvironment is capable of enhancing the tolerogenic ability of mature dendritic cells (DC) and promoting the secretion of immunosuppressive cytokines such as IL-27, IL-10 as well as IL-6. (e) Secreted galectin-1 impairs the tumor-killing effects of natural killer (NK) cells by attenuating the release of Granzyme B and Perforin. (f) Galectin-1 may give rise to macrophage polarization from M1 to M2 type with decreased biomarkers of M1 (iNOS, NO, and MHC-II) and elevated biomarkers of M2 (CD163, Arg1, and TGF-β). (g) Th1 and Th17 cells share a common glycan motif and are both susceptible to apoptosis induced by galectin-1. (h) Galactose-β1-4-N-acetylglucosamine ligands on the surface of Th2 cells, which were proved to be binding sites of galectin-1, were covered by sialic acid produced by α2-6 sialyltransferase (ST6Gal1), thus contributing to apoptosis resistance induced by galectin-1 binding. (i) Low concentrations of galectin-1 in the early stage of cancer can reprogram the tumor endothelium to elevate the expression of galectin-9 and PD-L1, thus mediating T-cell exclusion. (j) Endothelium-derived galectin-1 was found to act as a negative regulator limiting T cell rolling, capture as well as adhesion to endothelial cells.

Given that the suppressive tumor microenvironment is one of the major culprits accounting for the progression of cancer, galectin-1, an immune suppressive biomarker, has received a great deal of attention. As a versatile protein, galectin-1 is involved in multiple important life activities, so it is expressed in a variety of cell types, including stromal cells, mesenchymal stem cells, activated T cells, and many other types of cells, not just cancer cells. So, who plays a significant role in the formation of suppressive tumor immune microenvironment, tumor-derived or host-derived galectin-1? Banh et al. demonstrated in the galectin-1-deficient mice implanted Lewis lung carcinoma cells with high and low galectin-1 expression that host galectin-1 was responsible for promoting tumor immune privilege [62]. Furthermore, tumor galectin-1 is closely associated with hypoxia [63,64]. Zhao et al. revealed that the expression level of galectin-1 was mainly modulated by hypoxia-inducible factor-1α (HIF-1α), an oxygen-sensitive factor increased in hypoxic cancer cells [64]. Thus, galectin-1 production of cancer cells is elevated by the hypoxic tumor microenvironment, thereby exacerbating the suppressive immune microenvironment. Since its discovery of mediating apoptosis of T cells, a growing number of studies have shown that galectin-1 exerts immunomodulatory effects on multiple cells of the immune system, including macrophages, dendritic cells, natural killer (NK) cells, and many other cell types [65,66,67,68,69]. Therefore, the immune regulatory mechanisms of galectin-1 are highly complex, and there are several regulatory pathways that may work. The complex immunological roles of galectin-1 in the tumor microenvironment will be discussed below and illustrated in Figure 1.

### 5.1. Macrophages

Tumor-associated macrophages (TAMs) can display different polarization states, including anti-tumor M1-type macrophages and pro-tumor M2-type macrophages, in response to diverse stimulus signals [70,71]. Accumulating research has demonstrated that galectin-1 could regulate the function of TAMs. In vitro experiments, rat peritoneal macrophages pretreated with galectin-1 have led to the activation of L-arginase as well as the diminishment of inducible nitric oxide synthase (iNOS) and NO production induced by LPS [65]. Similarly, the inhibitory effect of galectin-1 on NO production was demonstrated in a *Yersinia enterocolitica* infection model [31]. Furthermore, Barrionuevo et al. showed that MHC-II expression, a biomarker of M1-type macrophages, was also reduced by galectin-1 [72]. Research of multiple myeloma revealed that serum galectin-1 was positively correlated to soluble CD163, indicating that galectin-1 might contribute to the activation of M2-like macrophages [73]. Therefore, galectin-1 may give rise to macrophage polarization from M1 to M2 type. This phenomenon was also reported by Van Woensel et al. when they knocked down galectin-1 in the tumor microenvironment of glioblastoma multiforme by applying siRNA-loaded chitosan nanoparticles [74]. Moreover, in the research by Chen et al., knocking down of *LGALS1* decreased the amount of M2 macrophages and attenuated the expression of immunosuppressive cytokines such as vascular endothelial growth factor A (VEGFA), C-C motif chemokine ligand 2 (CCL2) and transforming growth factor-β (TGF-β) [75].

In addition to modulating macrophage polarization, galectin-1 also exerts influences on monocytes, which are known as precursors of macrophages. Although galectin-1 shows no signs of inducing apoptosis of macrophages, it leads to apoptosis of monocytes [65,76]. Moreover, it was reported by Paclik et al. that Gal-1 could inhibit monocyte migration possibly by attenuating the expression of CD49d, which was an alpha subunit of integrin mediating migration [76]. Thus, galectin-1 may favor the immunosuppressive tumor microenvironment by exerting an important role in modulating central monocytes and macrophages.

### 5.2. Dendritic Cells

DCs exert multiple properties in the tumor immune microenvironment. Not only can DCs provide signals to activate T cells, but they also secrete cytokines to regulate the body’s immune response [77,78]. However, galectin-1 seems to perform paradoxical roles in modulating DCs, with the precise regulatory mechanisms remaining controversial. That galectin-1 could promote phenotypic and functional maturation of monocyte-derived DCs (MDDCs) with up-regulated CD86, CD40, CD83, and HLA-DR having been reported by Fulcher et al. They also found that galectin-1 increased the migratory ability of human MDDCs in vitro [66].

On the contrary, in the research by Thiemann et al., they found that galectin-1 inhibited immunogenic DC migration crossing lymphatic endothelial cells and extracellular matrix, rather than tolerogenic DC migration. In addition, they demonstrated this effect in a murine lymphedema model and showed a relation between migration and core 2 O-glycosylation of CD43 [79]. Furthermore, it was revealed by Ilarregui et al. that galectin-1 impaired the differentiation of immature DCs and enhanced the tolerogenic ability of mature DCs. Specifically, through the phosphorylation of signal transducer and activator of transcription (STAT) 3 in DCs, galectin-1 promoted IL-27 production of DCs, thus inhibiting IFN-γ production and proliferation of T cells and facilitating IL-10 secretion [80]. That maturation of DCs was suppressed by galectin-1 was also demonstrated in neuroblastoma and lung cancer [81,82]. In addition, lung cancer-derived galectin-1 was found to elevate the frequency of CD4+CD25+FOXP3+ Tregs besides diminishing Th1 cytokines and increasing IL-10 in MDDCs [82]. Interestingly, marrow-derived mesenchymal stem cells seem to take advantage of galectin-1 to impair the function of DCs [83]. Overall, the pro-inflammatory function of DCs was caused by galectin-1 in a high concentration (20 μM) [66], while the tolerogenic DCs was induced by lower concentrations that were much closer to the physiological galectin-1 level. Consequently, these seemingly paradoxical effects of galectin-1 may be dose-dependent.

### 5.3. T lymphocyte Cells

Since its discovery of inducing activated T cell apoptosis in 1995 [69], galectin-1 has been demonstrated to impair T cell adhesion to the extracellular matrix by inhibiting the re-organization of the actin cytoskeleton in these cells, thus thwarting cell migration towards inflammatory sites [84]. In addition, endothelium-derived galectin-1 was found to act as a negative regulator limiting T cell rolling, capture as well as adhesion to endothelial cells [85]. A recent study demonstrated that low concentrations of galectin-1 in the early stage of cancer could elevate the expression of galectin-9 and PD-L1 on tumor endothelial cells via activating STAT1, thus mediating T cell exclusion [86]. Moreover, it was reported that the presence of galectin-1 led to altered cytokine secretion, including the increase of IL-10 and the diminishment of IFN-γ [87,88]. Thus, galectin-1 may impair the function of T cells via impeding cell adhesion, migration, recruitment, and shifting the cytokine secretion pattern.

However, the precise mechanism of how galectin-1 modulates T cells remains controversial. Many studies have indicated that cell surface glycoproteins, including CD2, CD3, CD7, CD29, CD43, CD45, CD69, and CD95 as well as T cell receptor (TCR), could act as binding sites of galectin-1 [29,68,89]. Among them, T cell apoptosis mediated by CD45 upon binding to galectin-1 was found to be possibly associated with cell glycosylation status altered by the core 2 beta-1,6-N-acetylglucosaminyltransferase (C2GnT) [90]. Furthermore, in the presence of galectin-1, TCR signals that contribute to cell proliferation and IL-2 production was converted into apoptotic stimulation, which might relate to partial phosphorylation of the TCR-zeta chain [91,92]. CD7 and CD43 were also reported to play significant roles in apoptosis [93,94]. Meanwhile, the clustering of CD43 mediated by galectin-1 was found to inhibit T-cell migration [95]. In order to elucidate the intracellular downstream signals triggered by galectin-1 upon binding to glycoproteins on the surface of activated T cells, Rubinstein et al. reported that the activation protein-1 (AP-1) DNA-binding activity was an essential intracellular step [96]. Afterward, the activation of c-Jun N-terminal kinase (JNK) and phosphorylation of c-Jun was proved to be initiated by galectin-1, suggesting that JNK/c-Jun/AP-1 pathway appeared to act as a crucial role in response to galectin-1 stimulation [97]. Furthermore, it was shown that Fas, an apoptotic death receptor, could act as a binding site of galectin-1, whose recognition of Fas might activate caspase-8 [98,99]. The caspase cascade induced by galectin-1 might trigger morphogenetic changes and membrane depolarization of mitochondria, which was associated with ceramide production in the presence of p56lck and ZAP70 [99,100,101,102]. However, Hahn et al. showed that apoptosis induced by galectin-1 was elicited by a caspase-independent pathway accompanied by endonuclease G translocating from mitochondria to nuclei [103].

**Figure 2:**
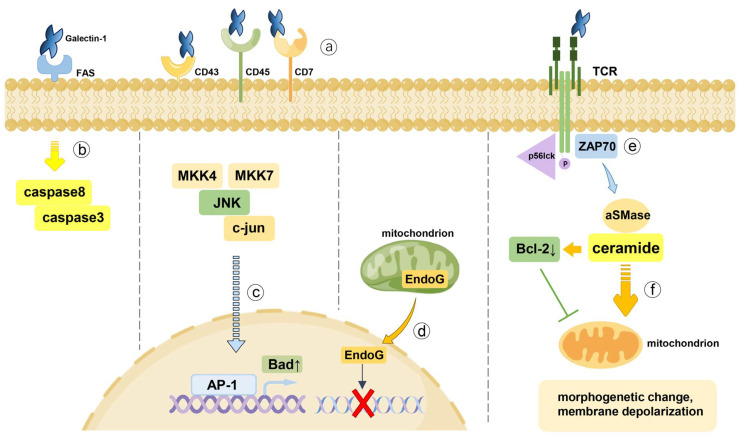
The mechanisms of T cell apoptosis induced by galectin-1. (a) Galectin-1 binding to CD7, CD43, and CD45 is a crucial step to mediate T cell apoptosis. (b) Fas (CD95) was proved to be a binding site of galectin-1, whose recognition of Fas might activate caspase-8 and downstream caspase-3. (c) Mitogen-activated protein kinase 4 (MKK4) and MKK7 were proved to be upstream activators inducing activation of c-Jun N-terminal kinase (JNK), which then mediating phosphorylation of c-Jun and enhanced activation protein-1 (AP-1) DNA-binding activity. (d) Endonuclease G, a mitochondrial endonuclease capable of triggering DNA degradation, was found to translocate from mitochondria to nuclei in the early stage of galectin-1-induced T cell death, which was considered a caspase-independent pathway. (e) TCR-zeta chain phosphorylated by p56lck may be associated with the coclustering of these two proteins induced by galectin-1. Thereafter, the partial phosphorylated TCR-zeta chain can act as a docking site for ZAP70 activation that leads to tyrosine phosphorylation. (f) Ceramide release depending on acid sphingomyelinase (aSMase) is a significant step during galectin-1 induced T cell apoptosis, which exhibits an intimate relation to anti-apoptotic Bcl-2 downmodulation and morphogenetic changes as well as membrane depolarization of mitochondria.

Overall, the effects of galectin-1 on T cells are multifaceted, with the exact mechanisms remaining obscure, and we presented the possible mechanisms in Figure 2. Given that galectin-1 exerts distinct actions on different lymphocyte cells, each of these cells will be discussed below.

#### 5.3.1. Cytotoxic T cells

In the research by Gandhi et al., Reed-Sternberg cell-derived galectin-1 in Hodgkin lymphoma diminished the infiltration, proliferation, and IFN-γ expression of Epstein-Barr virus-specific CD8+ T cells [104]. Moreover, galectin-1 was found to be expressed in exosomes secreted by numerous head and neck cancer cells, which could induce CD8+ T cells into a suppressive phenotype characterized by loss of CD27/CD28 expression [105]. Many other studies also reported that tumor-derived galectin-1 impaired the anti-tumor properties of cytotoxic T cells [62,81,106]. Interestingly, endogenous galectin-1, mainly produced by CD8+T cells, was also demonstrated to impair the cytotoxic function and proliferation of themselves in a murine prostate cancer model [107]. Therefore, galectin-1 appears to act as a negative autocrine regulator of activated CD8+ T cells, which might relate to the antagonistic action of the extracellular signal-regulated kinase (ERK) signaling induced by TCR [108]. Overall, as the final common effective tumor killer cells, cytotoxic T cells can be inhibited by multiple sources of galectin-1, thus establishing tumor immune privilege.

#### 5.3.2. Helper T Cells

It was reported that there were abundant Th2 cells in classical Hodgkin lymphomas, which was proved to be the result of galectin-1 stimulation [109]. Similarly, galectin-1 appeared to lead to a Th1/Th2 cytokine imbalance with impaired Th1 cytokine production and predominant Th2 cytokine profile in leukemic cutaneous T-cell lymphomas [110]. These results indicate that Th2 cells seem to resist the apoptotic effects of tumor-derived galectin-1. The research by Toscano et al. may explain this phenomenon. They found that galactose-β1-4-N-acetylglucosamine ligands on the surface of Th2 cells, which were proved to be binding sites of galectin-1, were covered by sialic acid produced by α2-6 sialyltransferase (ST6Gal1), thus thwarting the binding of galectin-1 [111]. The expression of ST6Gal1 in Th2 was much more than those in Th1, indicating that cell apoptosis induced by galectin-1 was related to the remodeling of cell surface glycoproteins by glycosyltransferases [111]. They also revealed that Th17 cells were susceptible to apoptosis induced by galectin-1 since they exhibited a common glycan motif as Th1 [111,112]. Furthermore, it was displayed in vitro that galectin-1 could thwart the differentiation of human Th17 cells via binding to CD69 [89]. At a concentration lower than its apoptotic concentrations, galectin-1 was able to promote IL-10 synthesis in both undifferentiated and polarized Th cells via its binding to CD45, which elevated IL-21 expression through transcriptional regulation of c-Maf/aryl hydrocarbon receptor pathway [112]. Thus, galectin-1 impairs the anti-tumor capacities of Th1 and Th17 cells and induces an immune suppressive microenvironment infiltrated with Th2 cells.

#### 5.3.3. Regulatory T Cells

That galectin-1 was expressed in mouse CD4+CD25+ Treg cells were initially detected through DNA microarray in 2002 [113]. Thereafter, Garín et al. reported that human as well as murine CD4+CD25+ Treg cells overexpressed galectin-1 that could be secreted into supernatants, and its expression was significantly elevated following activation. They also showed that the blockade of galectin-1 markedly impaired the inhibitory effects of these cells, indicating that galectin-1 played an essential role in maintaining T-cell tolerance mediated by CD4+CD25+ Treg cells [114]. Moreover, tumor-derived galectin-1 was demonstrated to attenuate the expansion of the CD4+CD25+FOXP3+ Treg cells in human Hodgkin lymphomas [109]. Similarly, knocking down galectin-1 expression in 4T1 cells led to a diminished proportion of CD4+CD25+Foxp3+ Treg cells within the tumor microenvironment as well as peripheral immune organs. The attenuation of galectin-1 also reduced the expression of the linker for activation of T cells (LAT) on Treg cells, thus disarming their inhibitory activities [115]. However, in a murine colorectal model, targeting galectin-1 did not lower the frequency of CD4+CD25+ Tregs in the tumor, spleen as well as tumor-draining lymph nodes, whereas the proportion and immunosuppressive properties of CD8+CD122+PD-1+ Tregs were reduced [116]. Accordingly, the effects of galectin-1 on Treg cells are context-dependent, with different tumor-derived galectin-1 may act differently on diverse Treg subsets.

### 5.4. Other Immune Cells

As a group of cells bearing immunosuppressive functions, myeloid-derived suppressor cells (MDSC) has been shown to be associated with galectin-1. It was reported that deletion of glioma-derived as well as human pancreatic stellate cell-derived galectin-1 led to a reduced number of tumor-infiltrating MDSCs [75,117,118].

In terms of natural killer cells, research by Baker et al. showed that glioma cells overexpressing galectin-1 could evade NK immune surveillance, whereas knocking down of galectin-1 culminated in tumor eradication prior to the initiation of the adaptive immune system [67]. A recent study indicated that mechanisms of sensitizing malignant glioma cells lacking galectin-1 to NK killing might relate to a decrease in the release of miR-1983, which could activate endogenous toll-like receptor 7 (TLR7) in plasmacytoid DCs as well as conventional DCs as a ligand. The binding of miR-1983 to TLR7 facilitates IFN-β secretion through the downstream MyD88-interferon regulatory factor (IRF) 5/IRF7 pathway, thus accentuating the tumor-killing effects of NK cells by releasing Granzyme B and Perforin [119]. Thus, galectin-1 favors tumor growth by impairing the anti-tumor effects of NK cells and facilitating the infiltration of MDSCs.

## 6. Galectin-1 Acts as a Two-Edged Sword in Autoimmune Diseases

A body of studies showed that galectin-1 played a protective role in diseases caused by the excessive immune response since this protein was able to induce apoptosis of T lymphocytes and impair the function of pro-inflammatory immune cells as well as facilitate infiltration of suppressive cells. However, there is growing evidence highlighting that galectin-1 can switch its role and be involved in the initiation and progression of inflammatory diseases. The dual roles of galectin-1 will be discussed separately in the following.

### 6.1. Promotive Role of Galectin-1 in Autoimmune Diseases

In normal testis, galectin-1 was detected to be expressed in germ cells as well as Sertoli cells, which was reported to act as an immunosuppressive factor stimulating the differentiation of tolerogenic DCs, indicating that galectin-1 was not a causative agent under normal conditions [120,121,122]. Nevertheless, Pérez et al. showed that galectin-1 deficient mice displayed a dramatic diminishment in the severity and incidence of experimental autoimmune orchitis (EAO) than wild-type mice, whereas the application of exogenous recombinant galectin-1 reduced the severity of this disease [123]. This seems to imply that exogenous galectin-1 limits the development of EAO, but endogenous galectin-1 promotes autoimmune inflammation. Similarly, research by Lei et al. revealed that TNFα and galectin-1 synergistically induced the expression of inflammatory cytokines including TNFα, IL-1α, IL-6 as well as monocyte chemoattractant protein-1 (MCP1) in Sertoli cells via activation of the mitogen-activated protein kinase (MAPK) signaling pathway, which might be a potential mechanism of the pro-inflammatory effects of galectin-1 [124]. In addition, an intimate relation of galectin-1 upregulation to osteoarthritic cartilage degeneration has been approved, accompanied by activation of NF-κB and elevated secretion of matrix metalloproteinases [125]. Overall, galectin-1 exerts dual roles in physiological conditions versus certain autoimmune diseases.

### 6.2. Protective Role of Galectin-1 in Autoimmune Diseases

Autoimmune diseases are usually elicited by an excessive immune response to auto-antigens. To repair the disorders of immune homeostasis and tolerance, Santucci et al. found that galectin-1 significantly improved the histopathologic and clinical features of 2,4,6-trinitrobenzene sulfonic acid-induced experimental colitis [126]. At a mechanistic level, the binding of galectin-1 to intestinal epithelial cells was facilitated in the presence of in vitro or in vivo inflammatory stimuli and fueled the secretion of tolerogenic cytokines including IL-10, IL-25, and TGF-β1 [127]. Furthermore, an increased proportion of CD4+Foxp3+ regulatory T cells as well as an altered Th17/Th1 profile was observed in the galectin-1 deficient mice, revealing how endogenous galectin-1 might regulate inflammation of the intestinal tract [128]. Additionally it was reported that galectin-1 was able to confer anti-inflammatory capacities to macrophages via inducing secretion of IL-10, thus ameliorating the murine dextran sodium sulfate-induced colitis [129].

In addition to inflammatory bowel diseases, exogenous and endogenous galectin-1 serve a protective function in arthritis. It was shown that galectin-1 treatment culminated in the alleviation of collagen-induced arthritis (CIA), accompanied by a decreased level of anti-collagen IgG as well as a skew toward Th2-type immune response [130]. Similarly, overexpression of galectin-1 was proved to induce apoptosis of antigen-activated T cells, therefore improving outcomes in rat CIA [131]. Additionally, galectin-1 deficient mice exhibited a faster onset of CIA as well as more severe disease progression, probably associated with enhanced secretion of IL-22 and IL-17 [132]. For Juvenile idiopathic arthritis characterized by mononuclear inflammatory infiltrates, galectin-1 appeared to confer amelioration through mononuclear apoptosis [133].

Experimental autoimmune encephalomyelitis (EAE) is a widely used animal model of multiple sclerosis (MS) that is characterized by immunologic disorders, axonal loss, and demyelination [134,135]. That administration of galectin-1 served to prevent the onset of EAE was first reported in 1990 [136]. Since then, a body of studies has revealed the intrinsic regulation by galectin-1. It was shown that galectin-1 led to dampened antigen-specific Th1 and Th17 responses, thus decreasing the clinical severity of EAE [111]. Furthermore, galectin-1 was demonstrated to act as a promoter endowing tolerogenic properties to DCs via the production of IL-27, and DCs could express galectin-1 to exert immunosuppressive capacity in EAE [80,137]. Of note, as one of the key cells mediating the process of multiple sclerosis, M1 microglia are inhibited by galectin-1 via thwarting the p38MAPK-, NF-κB, and CREB-dependent signaling pathways, with a concurrent decrease in the production of iNOS, TNF, and CCL2 [135]. Meanwhile, galectin-1 also facilitates microglial polarization towards M2-phenotype and augments their phagocytic capacity, resulting in the reduced demyelinated area as well as more efficient remyelination [138].

In a murine hepatitis model induced by concanavalin A (Con A), galectin-1 was shown to selectively eliminate the Con A-activated T cells, thus tending to act as a protective agent of human T cell-dependent liver disorders [139]. Given that galectin-1 mediated a glycan-binding dependent suppressive effect on antigen-activated T cells, type 1 diabetes caused by beta-cell-reactive T cells and experimental autoimmune uveitis mediated by Th1 could be alleviated following application of galectin-1 [140,141]. Moreover, in a recent study, aged galectin-1 deficient mice were demonstrated to exhibit similar spontaneously developed signatures of salivary gland inflammation in sjögren’s syndrome, indicating a protective action of age-dependent autoimmunity [142]. Of note, T cells from patients with systemic lupus erythematosus (SLE) exhibited significant resistance to galectin-1 binding, which might be due to glycosylation of cell surface glycan altered by the ratio of sialyltransferases and neuraminidase 1. Therefore, lowering the binding affinity between T cells and galectin-1 may lead to the onset of SLE [143]. Thus, the aforementioned studies suggested that galectin-1 conferred immunosuppression during the initiation and progression of autoimmune diseases.

## 7. Galectin-1: A Key Regulator of Allergic Inflammation

As described above, galectin-1 was able to impair the immunogenic property of lymphocytes and monocytes. Thus, it was conceivable that galectin-1 could lead to the resolution of T cell-mediated allergic inflammation, such as allergic contact dermatitis [144]. A recent study demonstrated that galectin-1 deficient mice exhibit more sustained skin inflammation with increased CD8+ T cell infiltration and IFN-γ secretion after the application of oxazolone. Of note, the proportion of neutrophils was also elevated in the skin [144]. Moreover, the application of galectin-1 tended to regulate eosinophils in the allergic airway inflammation [145,146], accompanied by decreased secretion of IL-25 in lung tissue and reduced concentrations of IL-17 and IgE as well as increased production of IL-10 in plasma [145,147]. Consistent with this, recombinant galectin-1 led to alleviation of pulmonary allergic response showed as inhibited mucus secretion and anergy of inflammatory cells [145]. Further in-depth research indicated that the effects of galectin-1 on eosinophils were dose-dependent. Ge et al. found that galectin-1 at lower concentrations inhibited the migration of eosinophils as well as the ERK signaling pathway, whereas exposure to higher concentrations led to phosphorylation of ERK and apoptosis of eosinophils with cytoskeleton disrupted [146]. However, in a murine allergic conjunctivitis model induced by ovalbumin (OVA), galectin-1 ameliorated this ocular allergy with attenuated production of Th2-type cytokines and decreased plasma anti-OVA IgE level but showed no relationship to eosinophil activation [148]. Additionally, through dampening the activation of mast cells in an IgE/FcεRI complex binding manner during allergen-specific immunotherapy (SIT), galectin-1 significantly alleviated the allergic inflammation and augmented the efficacy of SIT in a murine intestinal allergy model [149]. Overall, in addition to mediating the anergy of lymphocytes and monocytes, galectin-1 also exerts suppressive effects on mast cells as well as eosinophils to eradicate allergic inflammation.

## 8. Treatment Targeting Galectin-1

Given that galectin-1 is able to alleviate the excessive immune response, numerous studies have demonstrated that human recombinant galectin-1 can improve the prognosis of infection, organ transplantation, autoimmune diseases as well as allergic inflammation. However, in terms of diseases with a suppressive immune microenvironment, such as cancer, the expression of galectin-1 often contributes to the progression of the disease. Indeed, effective inhibitors of galectin-1 have been proved to display a significant anti-tumor effect. Currently, therapeutic agents can be mechanistically grouped into two categories: one class thwarts the binding of galectin-1 to cell surface carbohydrates and the other inhibits the expression of galectin-1. However, there is a class of inhibitors with their mechanism remaining unidentified. These three categories will be separately discussed in this section and are presented in Table 1.

### 8.1. Agents Inhibiting Galectin-1 Binding

As natural ligands of galectin-1, β-galactosides including lactulose, lactose as well as N-acetyllactosamine (LacNAc) show competitive inhibition of CRD-ligand binding [150]. However, the low inhibitory potency of these disaccharides limits their use [68,151]. By contrast, thiodigalactoside (TDG) exhibits an improved affinity for galectin-1 and is considered a non-metabolized disaccharide that is easy to produce [152]. Through antagonizing the immunosuppressive effects of galectin-1, TDG was reported to fuel the anti-tumor immune response stimulated by a vaccine in vivo [153]. In addition, Ito et al. showed that intratumoral injection of TDG alone was also capable of suppressing tumor progression by raising the infiltration of CD8+ lymphocytes and impairing tumor angiogenesis [150]. Moreover, new derivatives of TDG have been developed to enhance the binding affinity via increased arene–arginine interactions [151,154]. Among them, TD139 which is highly selectively binding to galectin-1 and galectin-3 has exhibited efficacy against Idiopathic Pulmonary Fibrosis in Phase Ib/IIa clinical trials (ClinicalTrials.gov: NCT02257177) [155,156].

Anginex is an artificial β-peptide that exhibits potent anti-angiogenesis properties [157]. Through binding to the β-sheet motif of galectin-1, this agent inhibits endothelial cells to impair microvessel formation, which contributes to the suppression of tumor growth [158,159]. However, this binding is non-specific since it also has an affinity for galectin-2, -7, -8, and -9 [160]. One possible explanation of how this peptide exerts biological functions is that interaction with galectins alters the equilibrium of galectin-ligand binding, but the specific mechanism remains obscure [160]. By contrast, OTX008 (0018), a calixarene compound designed based on the features of anginex, interacts with galectin-1 at a site that locates more distant from CRD than anginex [161,162,163]. Dings et al. found that OTX008 acted as an allosteric inhibitor thwarting the interaction of galectin-1 with cell surface carbohydrates [161]. Although this non-peptidic molecule has been demonstrated to normalize tumor vessels, attenuating tumor proliferation and invasion in various cancer cell lines and animal models, few studies have evaluated its role in humans [164,165,166]. There was a phase I trial investigating the effects of OTX008 therapy in advanced human solid tumors, but no results have been submitted to date (ClinicalTrials.gov: NCT01724320). Similar to OTX008, LLS30 was designed by Shih et al. as an allosteric inhibitor of galectin-1 that interacted with the carbohydrate-binding groove [167]. It has been reported that administration of LLS30 leads to the inhibition of cancer stem-like cells and invasion properties of hepatocellular carcinoma [168]. In addition, LLS30 confers an enhanced anti-tumor effect of docetaxel and suppresses the growth of castration-resistant prostate cancer [167].

Given that LacNAcs have been considered as galectin-1-binding determinants, peracetylated 4-fluoro-glucosamine (4-F-GlcNAc) that dampens the biosynthesis of LacNAcs favors the anti-tumor capacity of immune cells, thus attenuating tumor growth in melanoma and lymphoma [169]. Furthermore, a single-stranded DNA aptamer, AP-74 M-545, has been developed to antagonize galectin-1 with its high specificity and affinity. Tsai et al. showed that AP-74 M-545 circumvented the apoptosis of tumor-infiltrating T cells by blocking the CD45-galectin-1 binding in a murine lung cancer model [170].

Antibodies have the advantage of specific depletion of effector components. Since the neutralizing monoclonal antibody (mAb) of galectin-1 (8F4F8G7) was developed in 2012, a growing number of studies have proved that application of this antibody culminates in apoptosis resistance of T cells, attenuation of angiogenesis as well as tumor regression [171,172,173]. To further enhance the affinity, selectivity, and inhibitory potency of anti-galectin-1 mAb, recently Pérez Sáez et al. characterized a novel neutralizing antibody (Gal-1-mAb3) that suppressed the immunoregulatory and pro-angiogenic capacities displayed by galectin-1 [174]. Nevertheless, the application of mAbs may be restricted by their short half-life and high cost. To look for a cost-efficient and easy-designing alternative, an antibody-like polymeric nanoparticle (APN) was selected as a promising candidate for capturing and eliminating galectin-1 in tumor tissues [175]. Additionally, Femel et al. designed a murine galectin-1 vaccine (TRX–mGal1) that consisted of bacterial thioredoxin (TRX) fused to mouse galectin-1 (mGal1). By inducing the generation of endogenous antibodies against galectin-1, this recombinant vaccine significantly impaired the growth of melanoma in immunized mice [176]. As an alternative to traditional vaccines, a minigene DNA vaccine that contained DNA plasmid encoding immunogenic peptide fragment of galectin-1 also displayed effective activities against neuroblastoma [177].

### 8.2. Agents Inhibiting Galectin-1 Expression

Galectin-1 gene silencing by siRNA or lentivirus vector has been demonstrated to suppress tumor angiogenesis, improve the therapeutic benefits of temozolomide to human glioblastoma Hs683 cells and enhance the sensitivity of human lung adenocarcinoma to cisplatin [178,179]. However, how the siRNA can be delivered into the central nervous system tumor in a non-invasive way with limited systemic effects is a major concern for glioblastoma. To address this issue, Van Woensel et al. adopted concentrated chitosan nanoparticle suspensions by intranasal administration instead of intraventricular injection, which was capable of protecting the siRNA from RNAse degradation and reaching a higher percentage, thus effectively lowering the expression of galectin-1 [180]. They found that intranasal siRNA delivery synergistically improved the effect of anti-PD-1 therapy, accompanied by a decrease in infiltrated regulatory T cells and myeloid suppressor cells, increased infiltration of anti-tumor T cells, and tumor vascular normalization [74].

### 8.3. Agents with Unclear Mechanisms

Distinguished from the traditional galectin-carbohydrate binding domain, GM-CT-01 (Davanat), a galactomannan obtained from guar gum, is able to interact with galectin-1 at a site opposite to it [181]. It was shown that GM-CT-01 was capable of fueling IFN-γ secretion by CD8+ and CD4+ tumor-infiltrating lymphocytes (TIL) [182]. The Phase I and Phase II pre-clinical trials proved that GM-CT-01 was a non-toxic agent that improved the oncologic outcomes combined with 5-Fluorouracil (ClinicalTrials.gov: NCT00054977 and NCT00110721). In addition, there is an interventional Phase I/II Study to determine whether administration of GM-CT-01 leads to efficient cytotoxicity of TIL following peptide vaccination in Melanoma (ClinicalTrials.gov: NCT01723813).

Similarly, GR-MD-02 (Belapectin) is a galactoarabino-rhamnogalacturonan polysaccharide that can act as a galectin antagonist [156,183]. As GM-CT-01, GR-MD-02 also binds to galectin-1 as well as galectin-3 and exerts an anti-fibrosis effect in a murine model of Non-alcoholic steatohepatitis (NASH) as well as rat liver fibrosis model induced by thioacetamide [184,185,186]. Indeed, a number of clinical trials have shown that GR-MD-02 was well tolerated and was capable of reversing human fibrotic diseases such as NASH and Non-alcoholic fatty liver disease (ClinicalTrials.gov: NCT02421094, NCT02462967, and NCT01899859) [187,188,189]. Moreover, GR-MD-02 combined with an agonist anti-OX40 antibody was reported to accentuate anti-tumor immunity in tumor-bearing mice [190]. Of note, in a recent phase I study, GR-MD-02 plus anti-PD-1 agent (pembrolizumab) therapy was demonstrated to be safe and associated with favorable clinical responses to head and neck squamous cell carcinoma and metastatic melanoma (ClinicalTrials.gov: NCT02575404) [183]. However, how these polysaccharides bind to galectins and exert therapeutic efficacy remains controversial. Demotte et al. provided a possible explanation. They found that galectins were not detached from cells following binding to GM-CT-01, but increased TCR:CD8 colocalization, which improved the tumor-infiltrating lymphocytes (TIL) function [182]. However, the detailed mechanism needs to be further investigated.

**Table 1:** Treatments targeting galectin-1.

Agents	Materials	Mechanisms	Models/Trials	Refs
Thiodigalactoside	Disaccharides	Competitively inhibit galectin-1 binding	Melanoma, breast cancer	[149,151,152]
TD139	Derivatives of TDG	Competitively inhibit galectin-1 binding	Idiopathic Pulmonary Fibrosis (Phase Ib/IIa)	NCT02257177 [154,155]
Anginex	β-peptide	Alter the equilibrium of galectin-ligand binding	Murine ovarian carcinoma model	[156,157,158,159]
OTX008	Calixarene compound	Allosteric inhibitor of galectin-ligand binding	Human advanced solid tumors (Phase I)	NCT01724320 [160,161,162,163,164,165]
LLS30	Small molecule	Allosteric inhibitor of galectin-ligand binding	Prostate cancer, hepatocellular carcinoma	[166,167]
4-F-GlcNAc	Glycan	Dampen the biosynthesis of LacNAcs	Melanoma, lymphoma	[168]
AP-74 M-545	Single-stranded DNA aptamer	Impair galectin-ligand binding	Murine lung cancer model	[169]
8F4F8G7	Monoclonal antibody	Eliminate galectin-1 in tumor tissue	Kaposi’s sarcoma, prostate cancer	[170,171,172]
Gal-1-mAb3	Monoclonal antibody	Antibody with higher affinity and selectivity	-	[173]
APN	Antibody-like polymeric nanoparticle	Eliminate galectin-1 in tumor tissue	-	[174]
TRX–mGal1	Murine galectin-1 vaccine	Induce generation of endogenous antibody	Melanoma	[175]
Minigene DNA vaccine	DNA plasmid	Encode peptide fragment of galectin-1	Neuroblastoma	[176]
Intranasal siRNA	siRNA-loaded chitosan nanoparticles	Inhibit galectin-1 expression	Glioblastoma multiforme	[74,179]
GM-CT-01 (Davanat)	Galactomannan	Bind to galectin-1 at a site opposite CRD	Metastatic colorectal cancer	NCT00054977 and
			(Phase I and Phase II)	NCT00110721 [180]
		Increase TCR:CD8 colocalization	Melanoma (Phase I/II)	NCT01723813 [181]
GR-MD-02 (Belapectin)	Polysaccharide	Remain obscure	NASH, Non-alcoholic fatty liver disease	NCT02421094,
				NCT02462967 and
				NCT01899859 [186,187,188]
			Head and neck cancer, melanoma	NCT02575404 [182]
			combined with pembrolizumab (Phase I)	

## 9. Conclusions and Perspectives

Accumulating evidence shows that galectin-1 is a pluripotent regulator with resultant multiple functional manifestations in immune response (Table 2). Since various sugar-bearing compounds that act as ligands of galectin-1 are widely distributed across the membrane of a wide range of immune cells, galectin-1 plays a critical role in fate decisions of these immune cells, including cell apoptosis, polarization, proliferation, differentiation, recruitment as well as cytokine production, thus displaying a close association with a series of immune-related disorders (Table 3). However, it is the regulatory complexities of galectin-1 that interferes with our ability to explore comprehensive interactions and mechanisms. For example, galectin-1 binding to CD7, CD43, CD45, TCR as well as Fas are all involved in the apoptotic process of T cells, accompanied by activation of both caspase-dependent and independent pathways. To address these controversies, additional studies are required to clarify whether distinct cell types and states contribute to different apoptotic mechanisms, and which may play a dominant role. Moreover, compared with lymphocytes, there is a lack of research on the mechanisms of galectin-1 regulating innate immune cells, including how galectin-1 induces the polarization of macrophages and how it exerts different influences on different types of DCs. Additionally, given that the targets of galectin-1 are expressed widely on immune cells, exploration of its exact role on newly defined immune cell subsets represents an intriguing prospect, as Cagnoni et al. have identified its regulatory effects on CD8+CD122+PD-1+ Tregs [116]. Targeting galectin-1 possesses a potent efficacy in immune-associated diseases from experimental data, such as cancer. However, there are no FDA-approved agents available in clinics. Thus, it will be important to improve the target specificity as well as affinity and reduce the impact of galectin-1 blocking therapy on other immune cells.

That galectin-1 is a critical determinant of T cell apoptosis has been demonstrated since 1995, leading to substantial attention to the immunosuppressive properties of this protein. However, only in recent years have studies shown that galectin-1 appears to perform a pro-inflammatory role in certain diseases, suggesting the dual effects of galectin-1. Although the specific mechanisms remain controversial, we can conclude the possible causal factors based on the research discussed above. For pathogen-induced inflammation, galectin-1 binding to distinct microbes can either culminate in attenuation or promotion of infection, indicating that the effects of galectin-1 may depend on the microbial species, and the timing of microbial exposure to galectin-1 also contributes to different results. In addition, galectin-1 usually leads to the maintenance of immune tolerance in physiological status, whereas promoting the progression of inflammation in pathological conditions (such as sepsis, EAO, osteoarthritis, and the presence of specific cytokines). Moreover, the intrinsic biochemical parameters of galectin-1, including redox homeostasis and the monomeric or dimeric state, have an impact on its function. Finally, the stage of the inflammatory response, activation status of immune cells, glycosylation state of cell surface carbohydrate-bearing compounds and the dose of galectin-1 also perform a significant role. Thus, elucidation of how galectin-1 exerts paradoxical roles is worthy of further study to comprehensively understand the function of galectin-1 and search for safer and more effective therapies.

**Table 2:** The dual effects of galectin-1 in the immune response.

	Disease/Disease Model	Mechanisms	Effects	Refs
Pro-inflammation	Sepsis	Inhibit the unfavorable role of CD45 in endotoxin shock	Worsen lethal inflammation	[28]
	Infection of Dengue virus, influenzavirus, and NiV	Bind to certain envelope glycoproteins such as DENV-1, NiV-F, and NiV-G	Anti-infection	[48,49,50,51]
	Experimental autoimmune orchitis	Induce apoptosis of germ cells Synergistically enhance TNFα-induced inflammatory cytokine expression	Disease progression	[122,123]
	Osteoarthritis	Activate the NF-κB pathway and elevate secretion of matrix metalloproteinases	Disease progression	[124]
Anti-inflammation	Infection of *Yersinia enterocolitica* and *Tropheryma whipplei*	Attenuate production of IFN-γ, IL-17, TNF, NO; protect YOPs from trypsin digestion; facilitate bacterial cell entry	Pro-infection	[30,31,32]
	Murine acute inflammation model	Impair expression of adhesion molecules	Inhibit polymorphonuclear leukocyte migration	[38]
	Parasitic infection	Promote adhesion of parasites to host; fuel the immunotolerant circuits	Pro-infection	[40,41,42,43]
	Infection of HIV and NiV	Mediate virus adhesion to macrophages, CD4+ T cells, and epithelium	Pro-infection	[44,45,46,47]
	Graft versus host disease and graft rejection	Attenuate production of IL-2, IL-17, IFN-γ, and TNFα; suppress proliferation and alloreactivity of T cells	Prolong survival	[52,53,54,55,56,57,58,59,60]
	Cancer	Recruit suppressive immune cells; impair functions of cytotoxic leukocytes and alter the differentiation of naïve immune cells	Cancer progression	[65,66,67,68,69,70,71,72,73,74,75,76,77,78,79,80,81,82,83,84,85,86,87,88,89,90,91,92,93,94,95,96,97,98,99,100,101,102,103,104,105,106,107,108,109,110,111,112,113,114,115,116,117,118]
	Autoimmune diseases	Dampen antigen-specific T cell response; facilitate macrophage polarization towards M2; recruit suppressive immune cells	Disease remission	[125,126,127,128,129,130,131,132,133,134,135,136,137,138,139,140,141,142]
	Allergic inflammation	Dampen activation of mast cells and eosinophils and secretion of inflammatory cytokines	Disease remission	[143,144,145,146,147,148]

**Table 3:** Immunomodulatory effects of galectin-1 on immune cells.

	Immune Cells	Effects	Conditions	Refs
Pro-inflammation	Neutrophil	Promote ROS production	Primed neutrophil	[36]
		Facilitate migration	Physiological status	[39]
	MDDC	Promote migration and maturation	High concentration (20 μM)	[66]
Anti-inflammation	Neutrophil	Attenuate ROS production	Pretreatment with galectin-1 before activation	[36,37]
		Inhibit migration	Acute inflammation	[38]
		Promoted phagocytosis by macrophages	Activated neutrophil	[34,35]
	Eosinophil	Inhibit migration; induce apoptosis	-	[144,145,146]
	Mast cell	Inhibit activation	Bind to IgE/FcεRI complexes	[148]
	Macrophage	Suppress iNOS and NO production; induce polarization towards M2	-	[31,65,72,73,74,75,137]
	Monocyte	Inhibit migration; induce apoptosis	-	[65,76]
	DC	Inhibit migration and maturation of immunogenic DC	Low concentration	[79,80,81,82]
	CD8+ T, Th1 and Th17	Induce apoptosis; impair function and infiltration	Activated state	[89,103,104,105,106,107,108,109]
	Naïve Th cell	Promote suppressive cytokine secretion	Lower than apoptotic concentration	[111]
	Th2 cell	Resist apoptotic effect of galectin-1	-	[110]
	Regulatory T cell	Maintain suppressive function; promote infiltration	-	[112,113,114,115]
	NK cell	Impair cytotoxic property	-	[67,118]
	MDSC	Promote infiltration	-	[75,116,117]

## Data Availability

Not applicable.

## Abbreviations

CRD	Carbohydrate recognition domain
IFN-γ	Interferon-γ
IL	Interleukin
TNF	Tumor necrosis factor
NO	Nitric oxide
NF- kB	Nuclear factor kB
Th	T helper
PS	Phosphatidylserine
ROS	Reactive oxygen species
fMLP	N-formyl-methionyl-leucyl-phenylalanine
PMA	Phorbol myristate acetate
LPS	Lipopolysaccharide
NiV	Nipah virus
DENV-1	Dengue virus type 1
DC	Dendritic cell
Treg	Regulatory T cell
GVHD	Graft versus host disease
HIF-1α	Hypoxia inducible factor-1α
NK	Natural killer
TAMs	Tumor associated macrophages
iNOS	Inducible nitric oxide synthase
VEGFA	Vascular endothelial growth factor A
CCL2	C-C motif chemokine ligand 2
TGF-β	Transforming growth factor-β
MDDCs	Monocyte-derived DCs
STAT	Signal transducer and activator of transcription
TCR	T cell receptor
C2GnT	Core 2 beta-1,6-N-acetylglucosaminyltransferase
AP-1	Activation protein-1
JNK	c-Jun N-terminal kinase
ERK	Extracellular signal-regulated kinase
ST6Gal1	α2-6 sialyltransferase
LAT	Linker for activation of T cells
MDSC	Myeloid-derived suppressor cells
TLR7	Toll-like receptor 7
IRF	Interferon regulatory factor
EAO	Experimental autoimmune orchitis
MCP-1	Monocyte chemoattractant protein-1
MAPK	Mitogen-activated protein kinase
CIA	Collagen-induced arthritis
EAE	Experimental autoimmune encephalomyelitis
MS	Multiple sclerosis
Con A	Concanavalin A
SLE	Systemic lupus erythematosus
OVA	Ovalbumin
SIT	Allergen-specific immunotherapy
LacNAc	N-acetyllactosamine
TDG	Thiodigalactoside
4-F-GlcNAc	4-fluoro-glucosamine
mAb	Monoclonal antibody
APN	Antibody-like polymeric nanoparticle
TRX	Thioredoxin
mGal1	Mouse galectin-1
NASH	Non-alcoholic steatohepatitis
TIL	Tumor Infiltrating Lymphocytes
